# Coronary Artery Calcium Score - A Reliable Indicator of Coronary Artery Disease?

**DOI:** 10.7759/cureus.20149

**Published:** 2021-12-03

**Authors:** Devarashetty Shreya, Diana I Zamora, Gautami S Patel, Idan Grossmann, Kevin Rodriguez, Mridul Soni, Pranay K Joshi, Saawan C Patel, Ibrahim Sange

**Affiliations:** 1 Research, Gandhi Medical College and Hospital, Secunderabad, IND; 2 General Medicine, Universidad de Ciencias Medicas, San José, CRI; 3 Internal Medicine, Pramukhswami Medical College, Karamsad, IND; 4 Research, Medical University of Silesia, Katowice, POL; 5 Research, Universidad Americana (UAM), Managua, NIC; 6 Research, Shri Lal Bahadur Shastri Government Medical College and Hospital, Mandi, IND; 7 Research, Byramjee Jeejabhoy Medical College and Civil Hospital, Ahmedabad, IND; 8 Research, California Institute of Behavioral Neurosciences & Psychology, Fairfield, USA; 9 Research, Karamshi Jethabhai Somaiya Medical College and Research Centre, Mumbai, IND

**Keywords:** atherosclerosis, myocardial infarction, calcium scoring, agatston score, cac score, cad, coronary heart disease, coronary artery calcification, atherosclerotic cardiovascular disease

## Abstract

Coronary artery disease (CAD) is caused by atheromatous blockage of coronary vessels leading to acute coronary events that usually occur when a plaque ruptures and a thrombus forms. CAD is a known cause of significant cardiovascular events, accounting for more than 50% of the deaths in western countries, and most of the patients with CAD remain asymptomatic. The coronary artery calcium (CAC) score has been created as a measure of coronary atherosclerosis. This article has compiled various studies that conclude the clinical relationship between coronary artery calcium and the development of cardiovascular (CV) events by using the CAC score as a reliable indicator of CAD. This article has reviewed the pathophysiology and risk factors of CAD, along with various methods of CAC scoring. It also underlined the reliability of CAC scoring for early detection of CAD in asymptomatic individuals. We emphasized the importance of age-dependent risk factor analysis combined with practical screening tools like CAC scoring for early diagnosis of CAD can help direct the treatment and prevent deaths in asymptomatic individuals.

## Introduction and background

Atherosclerosis is the build-up of plaque in arteries that can constrict and impede blood flow. Coronary atherosclerosis, also known as coronary artery disease (CAD), refers to the building up of plaque in the main arteries supplying the heart muscle, leading to decreased blood flow. This plaque calcifies and ruptures over a period leading to thrombotic consequences [1]. CAD is one of the significant cardiovascular disorders that affect people all over the world. In both developed and developing countries, CAD is responsible for one-third of mortality among persons over 35, with the percentage in Western countries nearing 50 percent [2]. Although there is an equal susceptibility of CAD in men and women, women tend to have a better risk profile at a younger age and vice-versa in old age [3]. Pre-test probability (PTP) of obstructive CAD has been determined for several decades based on symptom presentation and recognized cardiovascular (CV) risk factors such as hypertension, hyperlipidemia, family history, and smoking. Many people have major adverse cardiac events (MACEs) despite being categorized as low risk based on traditional risk factors. These risk factor assessment scores only predict 65-80 percent of future cardiac events [4]. CAD is a complex illness, and early diagnosis of high-risk patients is critical since specific interventions can reduce clinical events and death significantly [5]. Coronary artery calcium (CAC) has been used as a diagnostic marker of atherosclerosis since the 1940s [6]. Janowitz et al. proposed a technique for measuring the amount of arterial calcification based on a specific score that measured the densities and extent of calcium in coronary arteries using Computed tomography (CT), which became known as the Agatston score, in 1991 [7]. A CAC score examination is a non-invasive examination of the coronary arteries in which the amount of calcium in the coronary arteries is determined using cardiac CT. Coronary calcium is exclusively the outcome of coronary atherosclerosis, with the exception of individuals with renal insufficiency, who may also develop medial calcification [8]. The quantity of calcium in the vessels is generally proportional to the amount of atheroma in the coronary vessels. The CAC score, which is determined using cardiac computed tomography, can assist in cardiovascular risk assessment and, as a result, clinical decision-making. According to the European Guidelines on heart disease prevention in clinical practice, the CAC score can be used to predict heart disease risk in asymptomatic adults at intermediate risk [8-10].

This review article aims to: 1) underline the pathogenic mechanism and associated risk factors involved in developing CAD and CAC; and 2) to explore the clinical relationship between coronary calcium and the development of CV events in asymptomatic individuals at greater risk of experiencing future cardiovascular events by using the CAC score as an effective screening tool.

## Review

Pathophysiology of CAD

**Figure 1:**
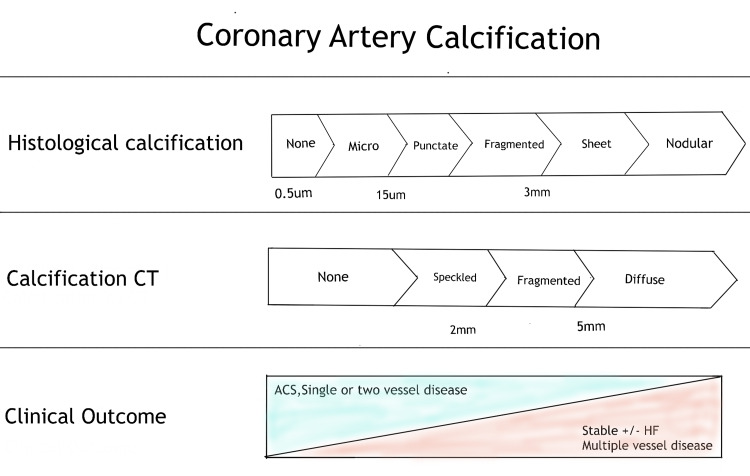
Coronary artery calcification CT - computed tomography, ACS - acute coronary syndrome, HF - heart failure

Coronary artery disease is typically caused by atheromatous constriction of the vessel and subsequent blockage. From young adulthood onwards, early atheromatous plaque (derived from the Greek word; *Athera *- porridge and *Oma *- lump) is evident. A mature plaque comprises two components that are macrophages and smooth muscle cells, each of which belongs to a particular cell lineage [11]. The necrotic "foam cells" - monocyte-derived macrophages that move into the intima and absorb lipids - are the primary source of the lipid core. Smooth muscle cells migrate in the vascular wall from the media into the intima, where they multiply and change their phenotype to create a fibrous capsule around the lipid core, forming the connective tissue matrix [11]. Calcification of the coronary arteries occurs in tandem with the progression of severe atherosclerosis. Calcification usually begins as micro-nodules (0.5 to 15.0 μm) and later progress to larger calcium particles which form sheets like deposition (>3mm) in the arteries. These changes appear to take place concurrently with plaque evolution (Figure 1) [12]. Coronary artery stenosis of more than 50% or a reduction in the cross-sectional area by 80% usually leads to angina on exertion. Intimal damage causes thrombus development by causing denudation of the thrombogenic matrix or lipid pool. Occlusion is more complete in acute myocardial infarction than unstable angina, where arterial occlusion is frequently partial. Acute coronary events usually occur when a plaque ruptures and triggers the formation of a thrombus [11]. 

**Figure 2:**
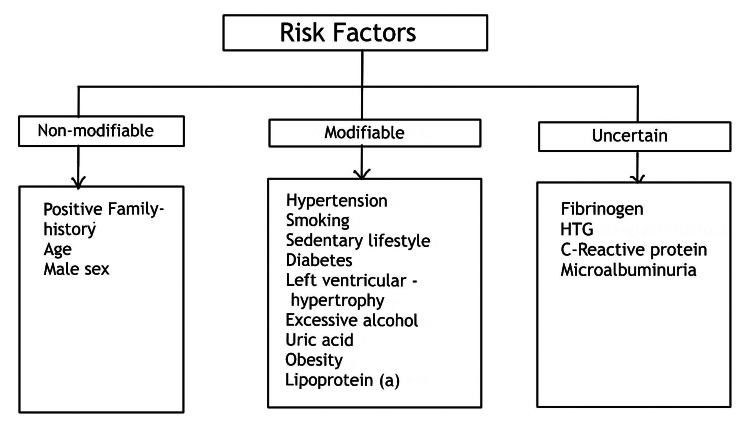
Risk factors associated with the development of CAD HTG - hypertriglyceridemia, CAD - coronary artery disease

Various factors have a role in the onset and advancement of calcification, which vary depending on the stage of plaque and the surrounding environment. Although it is usually associated with aging, various factors, including numerous associated risk factors that can accelerate the calcification in atheromatous plaques like environmental, genetic, family history, and lifestyle accounting for early CAD. Following are the associated risk factors (Figure 2) [13].

Here are inferences from studies stating the influence of risk factors on CAC: females experience a 10 to 15-year delay in the development of atherosclerosis compared to males, which is largely due to estrogen's preventative effects throughout the premenopausal years [14]. Hyperlipidemia has both direct and indirect effects on vascular calcification [15]. Diabetes mellitus with abnormal glucose metabolism can directly promote vascular cell calcification, and insulin can inhibit it [16,17]. And adipose tissue-derived factors regulate vascular calcification; adiponectin slows calcification, and leptin accelerates the process of vascular calcification [18,19].

​​Coronary artery calcium (CAC)

CAC is a highly accurate marker of coronary artery disease. Based on single-center and multicenter clinical and population-based studies with short-term and long-term outcomes, CAC scoring has emerged as a readily accessible, reliable, and efficacious means of assessing the risk of major cardiac events, especially in asymptomatic people planning for primary prevention interventions such as aspirin and statins [8]. In asymptomatic groups with a wide range of baseline risk, CAC testing is cost-effective [8]. Vascular calcification was formerly thought to be an inevitable, natural aging process. With medical advancements, atheroma calcification is now recognized as an active pathological process. Ectopic bone growth is a common feature of atherosclerosis, causes calcification of the coronary arteries [15]. The process is influenced by developmental, inflammatory, and metabolic variables. Master transcription factors including Msx2, Runx2, Osterix, and Sox9 and influential osteogenic differentiation factors like bone morphogenetic proteins (BMPs) two and four have been linked to vascular calcification. Matrix Gla protein is a BMP inhibitor that is abundant in calcified human arteries. The expression of both anti and pro-osteogenic elements in CAC demonstrates how tightly this process is controlled [20]. Many oxidative stress mediators are linked to calcification. Lipid oxidation produces pro-osteogenic mediators such as minimally altered low-density lipoprotein (LDL) and oxidized phospholipids [21]. Inflammation is induced by apolipoproteins and oxidized phospholipids in the vasculature, which is required for the development of vascular calcification and atherosclerosis [22,23]. Radiography, computed tomography (CT), and intravascular imaging can all be used to identify these calcification sheets and fragments [11].

The CAC score is calculated using 3 mm CT slices with no overlapping or gaps, limited to the cardiac region, acquired prospectively in synchrony with the electrocardiogram at a predetermined moment in the R-R interval, usually in the mid/late diastole, and without the use of intravenous contrast medium [24,25]. Calcification is defined as areas of at least 1 mm of hyper attenuation with > 130 Hounsfield units (HU) or fewer than three consecutive pixels [26]. CAC may be detected using a variety of imaging modalities. However, non-contrast, electrocardiographic gated - multidetector computed tomography (MDCT) and electron beam computed tomography (EBCT) are the most commonly used in outpatient settings. The CAC score was first investigated using EBCT, and much of the scholarly literature at the time was based on this method [24]. Later MDCT has become the modality of choice for CAC evaluation. As a result, electron beam computed tomography is now almost non-existent [24].

There are various methods for the assessment of CAC scores. The Agatston method, calcium volume determination, and calcium mass score are the three basic approaches for calculating the CAC score [25-28]. The first two are the most extensively used, notably the Agatston technique, which is cited in most population databases and articles and is, therefore, the most widely employed in clinical practice. Calcium density and calcium content measurements are more consistent [29].

The Agatston Method

The Agatston method uses the weighted sum of lesions with a density above 130 HU. It multiplies the area of calcium by a factor related to maximum plaque attenuation (130-199 HU, factor 1; 200-299 HU, factor 2; 300-399 HU, factor 3; and 400 HU, factor 4) [30,31]

Calcium Volume Score

This method is the most reliable and repeatable [30]. It is calculated by multiplying the number of calcified voxels by the volume of each voxel, considering all voxels with an attenuation more significant than 130 HU. However, depending on the position of the plaque in the axial slice acquired, this method is particularly sensitive to partial volume (especially in plaques with high attenuation) and subject to variability between tests [30,31].

Relative Calcium Mass Score

The relative calcium mass score is calculated by multiplying the calcified plaque's mean attenuation by the plaque volume in each image, reducing the variation caused by partial volume. A correction factor based on water attenuation is used to calculate the absolute calcium mass score [30,31].

The Agatston CAC score can be interpreted and classified by adjusting values for patient age, gender, and ethnicity and calculating distribution percentiles in the general population using several population databases (Table 1).

**Table 1:** Agatston coronary artery calcium scoring CAC – coronary artery calcium

CAC score (Agatston method)	Risk analysis	Clinical correlation
0	Absent/ No risk	Low risk of future cardiovascular events.
1-10	Minimal	Minimal atherosclerosis may be present with a low risk of future cardiovascular events.
11-100	Mild	There is likely mild to minimum coronary artery stenosis. A mild risk of coronary artery disease exists.
101-400	Moderate	Reasonable amount of plaque can be confirmed. Has a moderately increased risk of future cardiovascular events.
>400	High	A high coronary calcium score corelated with a significant risk of having a cardiovascular event (such as myocardial ischemia) in near future.

Coronary artery calcium score and coronary vascular disease

**Figure 3:**
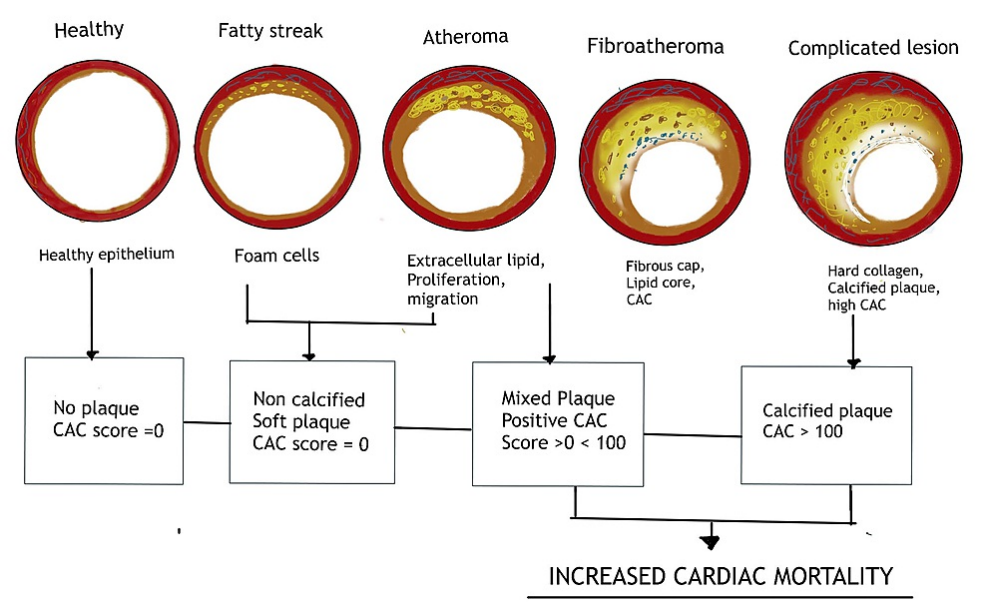
Coronary artery calcium scoring and coronary vascular disease CAC - coronary artery calcium

Asymptomatic individuals with no associated risk factors and a calcium score of zero are highly unlikely to have any significant luminal obstruction or atheromatous plaque. They are also at very low risk of any cardiovascular events within the next two to five years [32]. Positive (non-zero) CAC scores, on the other hand, indicate the existence of coronary atherosclerotic plaque, and rising values are linked to increased coronary heart disease (CHD) risk (Figure 3) [33].

The Multi-Ethnic Study of Atherosclerosis (MESA) started in 2000 with a prospective multi-center cohort sample of 6,814 men and women aged 45 to 84. Approximately 38% of the recruited participants were white, 28% were African American, 22% were Hispanic, and 12% were Asian. MESA used EBCT scanners in three locations and MDCT systems in three locations [34]. For both EBCT and MDCT, CAC distributions were similar [34]. CAC was shown to differ by ethnicity, with whites having a higher incidence than the other three ethnic groups [34]. Risk factor differences did not entirely explain variations in CAC distributions between ethnicities, indicating that other variables must account for part of the variation in CAC distributions. MESA offered estimated curves for the 50th, 75th, and 90th percentiles of calcium throughout age, allowing users to see briefly what an approximate percentile signifies for a certain patient (Table 2) [34]. Similarly, Framingham Heart Study (FHS) in 2005 incorporated a CAC assessment by MDCT to the Framingham Offspring and Third Generation cohorts' examinations. Although the FHS only includes white men and women, the CAC >0 and CAC >100 distributions were remarkably comparable to those previously reported from MESA. A new analysis of CAC data looked at whether information on the distribution of CAC and coronary dominance, as found by MDCT, helped predict incident CHD in addition to the standard Agatston score. After multivariable adjustment, the study concluded that the number of coronary arteries with CAC and CAC presence in the proximal dominant coronary artery were substantially linked with major CV events over a median follow-up of seven years (Table 2) [35]. Also, the Coronary Artery Risk Development in Young Adults (CARDIA) research, the first prospective cohort to collect data on CAC among 2,831 patients aged 32 to 46, assessed CAC throughout follow-up. CAC >0 is not rare in this age group, according to CARDIA, especially when a risk factor is present. CAC highly predicted risk beyond established risk variables in these young people over a 10-year follow-up (Table 2) [36]. Dennis et al. conducted a prospective study in 263 patients (women aged 30-65 years and men aged 30-62 years) with chest pain and low-to-moderate risk of coronary artery disease. They performed a traditional emergency department (ED) chest pain evaluation as well as a CT CAC scan. 133 (51%) had a CAC score of zero. The absence of CAC was highly linked to the chance of experiencing noncardiac chest discomfort. Only one (1%) of the 133 patients with a CAC score of 0 developed cardiac chest discomfort. On the other hand, 30 (97%) of the 31 patients with cardiac chest discomfort revealed evidence of CAC on CT. According to the findings, CT CAC assessment is an effective supplement in evaluating people at low-to-intermediate risk and concluded that in this cohort, cardiac chest discomfort is highly unlikely due to the lack of or mild CAC (Table 2) [37]. Dekker et al. conducted a single-center observational cohort study of 1265 patients in the Netherlands from 2014-2016. Only individuals who had a coronary angiography (CAG) within 90 days before or after myocardial perfusion imaging (MPI) were included in this study. The final cohort consisted of 150 patients after excluding those who had previously undergone coronary artery bypass grafting (CABG) or percutaneous coronary intervention (PCI), as well as five individuals who had incomplete MPI data. CAC scores combined with MPI increase obstructive coronary artery disease diagnosis in people who have never had a revascularization procedure (Table 2) [38]. Table 2 lists the included studies of all different designs conducted between 2005 - 2019 that link the relationship between CAC score and CAD in asymptomatic individuals.

**Table 2:** Summary of studies of different designs conducted between 2005-2019 that link the relationship between CAC score and CAD in asymptomatic individual MESA- Multi-Ethnic Study of Atherosclerosis, CARDIA - Coronary Artery Risk Development in Young Adults, FSH - Framingham Heart Study, CAC - coronary artery calcium, CAD - coronary artery disease, CHD - coronary heart disease, CVD - coronary vascular disease, MPI - myocardial perfusion imaging

References	Study Year	Design	Sample population	Age of the participants	Percentage with CAC >0 at baseline examination	Conclusion
MESA [33]	2005	Prospective multicenter cohort	6,814	45-84, mean age: 62.2 ± 10.2	Men: 52%– 70%, women: 35%–45%	Beyond known risk factors, CAC reliably predicted cardiovascular risk in all four ethnic groups, with similar strength in all four ethnic groups..
Dennis et al. [36]	2010	Prospective study	263	30-62, mean age: 46	49%	On a five-year follow-up 1% of the 133 patients with a CAC score of 0 developed cardiac chest discomfort. The absence of CAC suggests an excellent long-term prognosis.
CARDIA [35]	2017	Prospective community-based study	5115	32-56, mean age 40.3		After surveillance for 30 years, it concluded that a CAC score of 100 or above was linked to a higher risk of mortality. Adults under the age of 50 who have any CAC found on a computed tomographic scan, even with extremely low scores, are at an increased risk of clinical CHD, CVD, and mortality.
FHS Study [34]	2017	Observational cohort study	3,238	Men >35, women >40, mean age 49 ± 10.9	Men: 40.5%, women: 20.6%	The presence and extent of coronary artery calcium (CAC) are associated with increased risk for cardiovascular events.
Dekker [37]	2019	Observational cohort study	1265	Mean age 67.6	94%	CAC scores combined with MPI increase the diagnosis of obstructive coronary artery disease in people who have never had a revascularization procedure.

Coronary artery calcium (CAC) monitoring has become a popular subclinical approach for predicting cardiovascular disease in asymptomatic people. The predictive value of CAC scanning in symptomatic people is less established. It has no predictive value in already symptomatic people and is associated with additional cost and radiation burden. Pursnani et al. conducted a prospective study in the United States in 2015 with 473 elderly population of age group 54±8 years, 53% men concluded that CAC score does not provide additional value beyond coronary computed tomography angiography (CCTA) for acute coronary syndrome (ACS) diagnosis in emergency department patients with acute chest pain. CAC=0 does not rule out ACS, and a high CAC score does not rule out CCTA interpretation in most patients [39]. Chang et al. performed a prospective observational cohort study in the emergency department of the Hospital of the University of Pennsylvania with a total of 1049 patients of medical age 48.1, 55% female, 63% African American concluded that coronary angiography calcium scores alone could not risk stratifying patients in the emergency department who may be suffering from acute coronary syndromes. The addition of the coronary angiography calcium score to coronary computed tomography angiography did not improve prognostic value [40]. These studies helped in concluding that CAC score has no significance in symptomatic patients.

Limitations 

CAC is a complicated disease condition. Although we were able to explore the pathophysiology and risk factors, we could not locate enough research to detail the impact of different pathogenic etiologies such as chronic renal disease and parathyroid abnormalities on atheroma calcification. In addition, utilizing the CAC score as an indication, we were able to demonstrate a clinical relationship between CAD and CAC in the aforementioned review study. Despite this, we were unable to determine how early identification affected the treatment result in these individuals.

## Conclusions

The clinical implication of this review article is to establish a clinical association between CAC and CAD. As previously stated, atheromatous constriction of the artery is the most common cause, and subsequent obstruction and the quantity of calcification of atheromatous plaque can predict the occurrence of CV events. The calcium score in the coronary arteries is a reliable predictor of coronary heart disease events. It has become a widely available, accurate, and dependable tool for determining the risk of major cardiovascular events, particularly in asymptomatic individuals. It is gaining popularity because of its high accuracy in predicting atherosclerotic cardiovascular disease risk in asymptomatic individuals with low to moderate atherosclerotic cardiovascular disease risk. In symptomatic people, however, this relationship is meaningless. By publishing this article, we hope that clinicians will be able to use CAC scoring as a valid screening tool for early diagnosis of CAD in asymptomatic people, allowing them to take the appropriate preventative actions to decrease CAD-related mortality and morbidity. Because of its ease of use and lack of invasiveness, it is hoped to be broadly used in clinical practice. Although we could establish a link between CAC score and CAD, additional research is needed to determine how useful this can be in avoiding future CV events and assisting in treatment.
